# *Pseudomonas aeruginosa* PAO1 outer membrane vesicles-diphtheria toxoid conjugate as a vaccine candidate in a murine burn model

**DOI:** 10.1038/s41598-022-26846-z

**Published:** 2022-12-24

**Authors:** Ehsan Zare Banadkoki, Iraj Rasooli, Tooba Ghazanfari, Seyed Davar Siadat, Mehdi Shafiee Ardestani, Parviz Owlia

**Affiliations:** 1 Department of Medical Microbiology, Faculty of Medicine, Shahed University of Medical Sciences, Tehran, Iran; 2 Department of Biology, Shahed University, Tehran, Iran; 3 Molecular Microbiology Research Center, Faculty of Medicine, Shahed University, Tehran-Qom Express Way, Tehran, 3319118651 Iran; 4 Department of Immunology, Faculty of Medicine, University of Shahed, Tehran, Iran; 5 Department of Mycobacteriology and Pulmonary Research, Pasteur Institute of Iran, Tehran, Iran; 6 Department of Radiopharmacy, Faculty of Pharmacy, Tehran University of Medical Sciences, Tehran, Iran

**Keywords:** Biotechnology, Immunology, Microbiology

## Abstract

*Pseudomonas aeruginosa* is an opportunistic pathogen considered a common cause of nosocomial infection with high morbidity and mortality in burn patients. Immunoprophylaxis techniques may lower the mortality rate of patients with burn wounds infected by *P. aeruginosa*; consequently, this may be an efficient strategy to manage infections caused by this bacterium. Several pathogenic Gram-negative bacteria like *P. aeruginosa* release outer membrane vesicles (OMVs), and structurally OMV consists of several antigenic components capable of generating a wide range of immune responses. Here, we evaluated the immunogenicity and efficacy of *P. aeruginosa* PA-OMVs (PA-OMVs) conjugated with the diphtheria toxoid (DT) formulated with alum adjuvant (PA-OMVs-DT + adj) in a mice model of burn wound infection. ELISA results showed that in the group of mice immunized with PA-OMVs-DT + adj conjugated, there was a significant increase in specific antibodies titer compared to non-conjugated PA-OMVs or control groups. In addition, the vaccination of mice with PA-OMVs-DT + adj conjugated generated greater protective effectiveness, as seen by lower bacterial loads, and eightfold decreased inflammatory cell infiltration with less tissue damage in the mice burn model compared to the control group. The opsonophagocytic killing results confirmed that humoral immune response might be critical for PA-OMVs mediated protection. These findings suggest that PA-OMV-DT conjugated might be used as a new vaccine against *P. aeruginosa* in burn wound infection.

## Introduction

*Pseudomonas aeruginosa* (*P. aeruginosa*) is a gram-negative opportunistic pathogen responsible for severe infections in various body organs, such as the urinary tract, surgical wounds, burns, and the lower respiratory tract^1^. Approximately 20% of nosocomial infections caused by gram-negative bacteria are caused by *P. aeruginosa*, especially in burn patients^2^. Burn wounds, as one of the main occurring skin or other tissue injuries, are severe causes of mortality and morbidity worldwide^3^. The World Health Organization estimates that more than 300,000 people die annually due to severe burns, most of which occur in low- and middle-income countries^4^. According to epidemiological research, in Iran, burn is the most common injury after road accidents in all trauma causes^5^. By compromising the integrity of the skin, burns weaken the body's natural defense against bacterial invasion^6^. Therefore, the burn wound facilitates a suitable place to enter and bacterial colonization, which intensification the systemic spread of it through the body^7^. In the presence of a deficient immune system, the burn infection becomes a life-threatening infection caused by pathogen strains. With increased antimicrobial resistance and the challenges in the discovery of new antibiotic classes, new therapeutic options are needed to successfully control antimicrobial resistant bacterial infections^8^. Vaccines represent one of the most promising options to control antimicrobial resistance. Effective vaccines would prevent infections caused by multi-drug resistant (MDR) bacteria. Vaccines have also been shown to significantly reduce antibiotic prescription and thus reduce antimicrobial resistance^9^. Infection of a burnt wound by antibiotic-resistant organisms, such as multidrug-resistant (MDR) and extensively drug-resistant (XDR) *P. aeruginosa*, has been reported as the leading cause of mortality and morbidity^10^. Remarkably, these strains possess considerable ability to survive in healthcare settings, particularly in burn care units. Since broad-spectrum antibiotics are widely used in these units, it leads to the continuous emergence of MDR and XDR *P. aeruginosa*^11–13^. Accordingly, antimicrobial therapy is often ineffective in infections caused by MDR and XDR-*P. aeruginosa*. A mortality rate of 13.5% has been reported in ventilation-associated pneumonia caused by *P*. *aeruginosa*^14^ whereas this bacterium constituted approximately 77% of mortality in burn patients during the past 25 years^15^. Therefore, developing new alternative prevention and treatment strategies is essential for treating burn wounds infected by *P. aeruginosa*. Despite the efforts made, there is still no vaccine available against this bacterium^16^, but vaccine design as a cost-effective active and passive immunization technique may thereby prevent *P. aeruginosa* infections and limit the emergence of MDR and XDR *P. aeruginosa* strains^17^. Various vaccines and several monoclonal antibodies were developed during the past decade for *P. aeruginosa* infections^18^. In previous studies, several antigens of *P. aeruginosa* were used as vaccine candidates, including lipopolysaccharides (LPS), outer membrane proteins (OMPs), such as OprF, OprI, and exopolysaccharide like PSL^19,20^ and extracellular component PcrV and flagellates; however, despite numerous efforts to develop an effective vaccine against *P. aeruginosa*, no vaccine has yet been approved for human use^21–24^. Outer membrane vesicles (OMVs) are an emerging technology for inducing anti-bacterial immunity. OMVs are exocytic vesicles produced naturally by many Gram-negative bacteria. They are thought to serve multiple functions from cell-to-cell communication, protein secretion, virulence and aiding in biofilm formation. OMVs have a lipid bilayer that contains lipopolysaccharide and cell surface proteins. They are good potential vaccine candidates because they can be taken up by mammalian cells, they are strongly immunogenic^25^ and proteins found in OMVs have a native conformation^26,27^. Recently developed vaccine candidates contain outer membrane vesicles (OMVs)^28,29^. By recognizing these vesicles, it was discovered that OMVs contain antigens such as phospholipid, peptidoglycan (PG), OMP, LPS, and components of membrane lipoproteins with periplasmic proteins that are capable of inducing a variety of immune responses^28,30^. We hypothesized that conjugating PA- OMVs -diphtheria toxoid (DT) as a carrier protein could induce a significant immune response and provide full protection against *P. aeruginosa* in the burn mice model because recent studies have shown that PA-OMVs could be used as a candidate antigen for vaccine development. Thus, this study evaluates the immunogenicity, and protective efficacy of PA-OMVs-DT conjugate as a candidate vaccine in mice burn models infected by *P. aeruginosa* strain PAO1.

## Results

### Characterization and morphology of PA-OMVs

**Figure 1:**
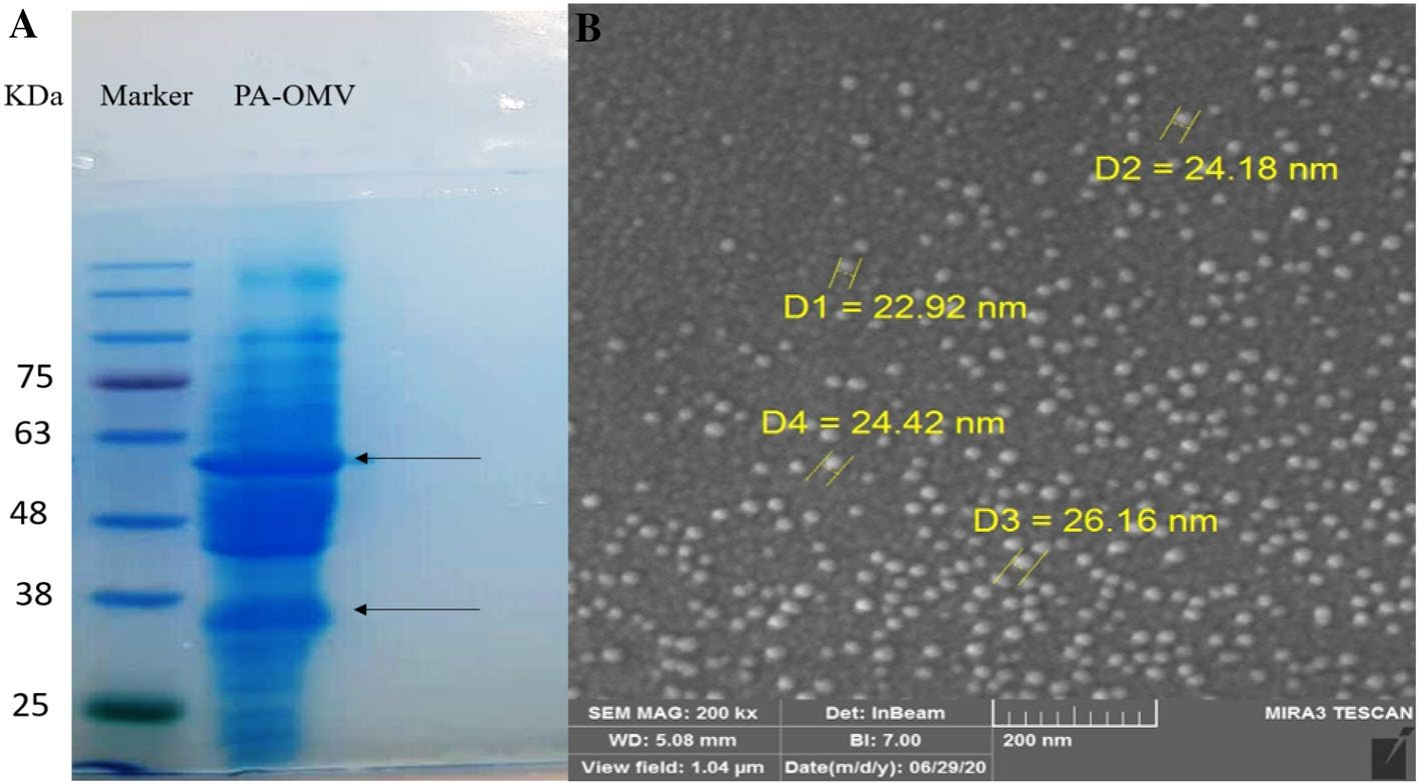
Characterization of PA-OMVs isolated from *P. aeruginosa*. (**A**) Coomassie brilliant blue-stained SDS–PAGE of PA-OMVs. (**B**) FE-SEM of PAO1 derived OMVs.

The pattern of bands is consistent with the size of these proteins as reported in a previous study^31^ (arrows in Fig. 1A and supplementary Figure 1). As shown in (Fig. 1B), The spherical PA-OMVs vesicles observed in Fig. 1B, ranged between 20 and 200 nm in size.

### Characterization and analysis of the PA-OMVs-DT conjugate

**Figure 2:**
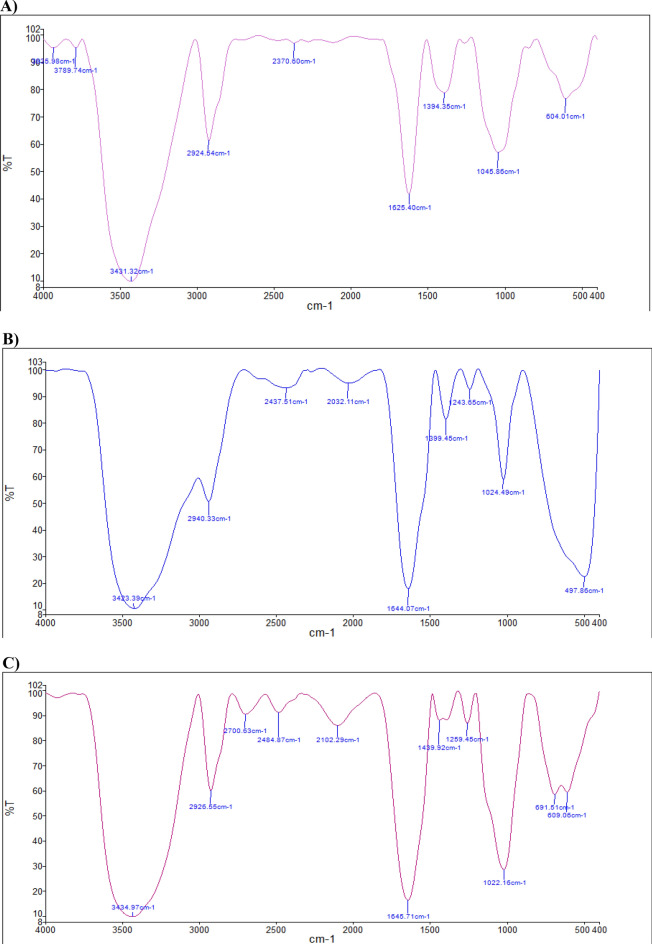

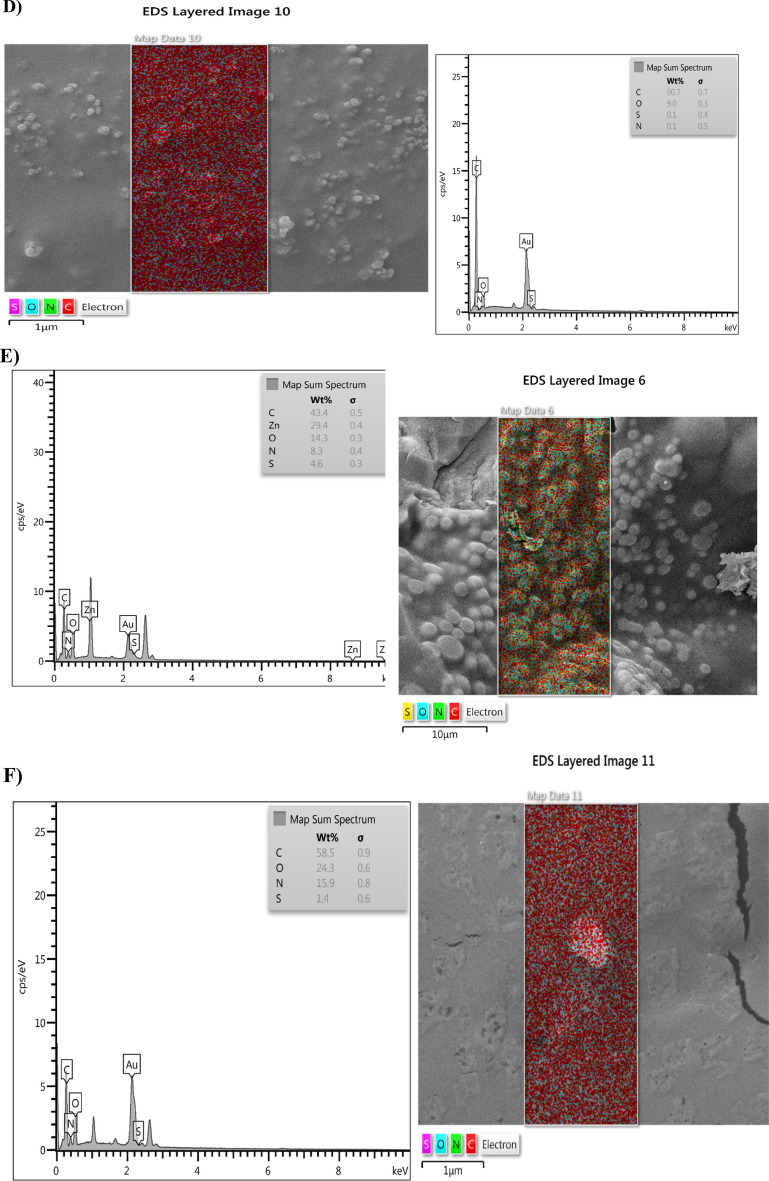

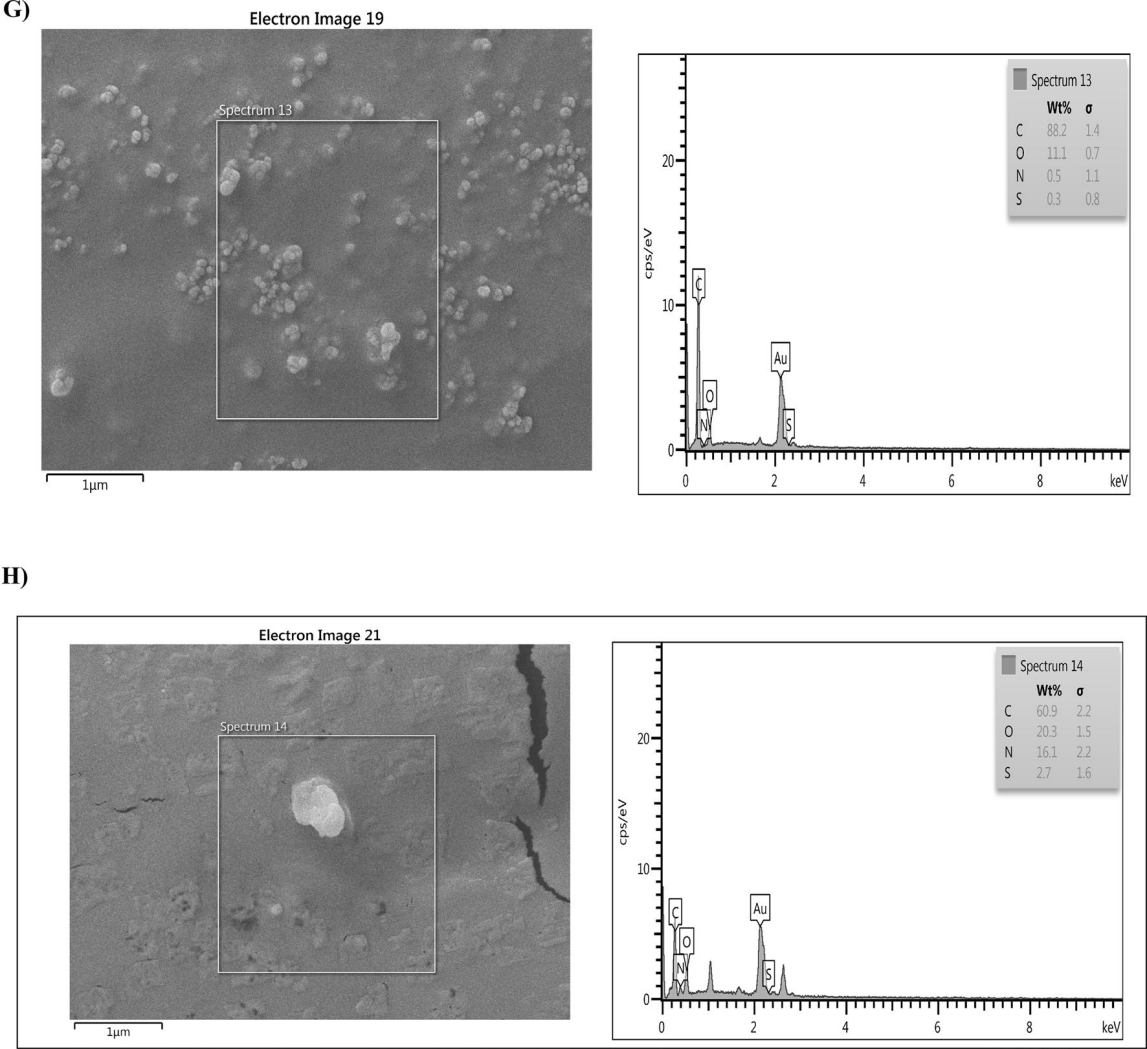
FTIR, Mapping, and EDAX structural analyses. (**A**) PA-OMVs, (**B**) DT, and (**C**) PA-OMVs-DT structures analyzed by FTIR. (**D**, **E**, and **F**) structures analysis of PA-OMVs, DT, and PA-OMVs-DT by Mapping analysis. (**G** and **H**) structural analysis of the PA-OMVs, and PA-OMVs-DT by EDAX.

The comparison of conjugated peaks by FTIR spectroscopy shows that OMVs-DT conjugate peaks in one-component samples had a slight numerical difference. Moreover, this peak in three molecules demonstrated the proper production of the planned conjugate. Amide bonds are prevalent in the structures of proteins (DT and PA-OMVs). Numerically, the value of this peak in the PA-OMVs molecule is 1625.40, in DT molecule is 1644.07, and the formed conjugate is equal to 1645.71 (Fig. 2A, B, and C). The changes in peaks indicated that the amide bonds formed in the PA-OMVs-DT conjugate confirmed the correctness of conjugation process. Moreover, the peaks assigned among 3434.97, 2926.55, and 1645.71 Cm^−1^ belonged to OH, CH, and C=O groups, respectively (Fig. 2C) were seen in three molecules and confirmed the proper production of the conjugate. The outcomes of mapping analysis are both qualitatively and quantitatively discernible. The composition of the components may also be defined as a percentage, and they can be represented in color.

MAPPING analysis (Fig. 2D, E, and F) shows that the conjugated component (OMV and DT) elements are also seen in the conjugate confirming the conjugation and formation of a new molecule.

The elemental composition maps based on EDAX analysis confirmed the existence of distinct elements in PA-OMVs-DT conjugate and conjugate components individually, as shown in (Fig. 2G and H). The findings revealed a minor difference in the percentages of conjugate and conjugate parts (numerically).

### PA-OMVs-DT conjugate vaccination and survival rate of burnt mice infected with PAO1

**Figure 3:**
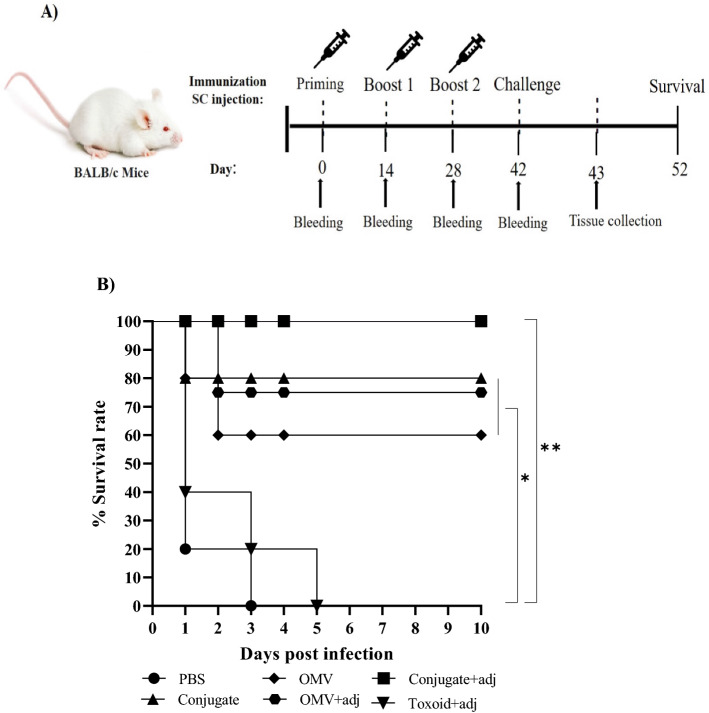
Experimental scheme of mice immunization and survival rate monitoring. (**A**) Schematic presentation of mice immunization. (**B**) The survival rates of immunized BALB/c mice (n = 10) infected with PAO1 (3 × 10^2^ CFUs) in the burn model were monitored twice a day for ten days, and rates were analyzed using the log-rank test method. ∗*P* < 0.05 and ∗∗*P* < 0.001.

Schematic presentation of mice immunization is illustrated in Fig. 3A. The mice in the PBS group all died within the first three days of infection. All the unchallenged burnt mice in the control group survived. Groups vaccinated with the PA-OMVs-DT + adj conjugated exhibited higher survival rates (100%) than all other groups, significantly protecting immunized mice against PAO1 infection. Furthermore, immunization with PA-OMVs-DT without adjuvant and PA-OMVs + adj yielded a protective efficacy of 80 and 75%, respectively, higher than the PBS group. As shown in (Fig. 3B), PA-OMVs without adjuvant immunized mice have a partial protective role against PAO1 infection (60%) compared to the control group. No survival was noted in the toxoid-adj group (Fig. 3B).

### Conjugated PA-OMVs vaccination protects mice with decreased bacterial burden

**Figure 4:**
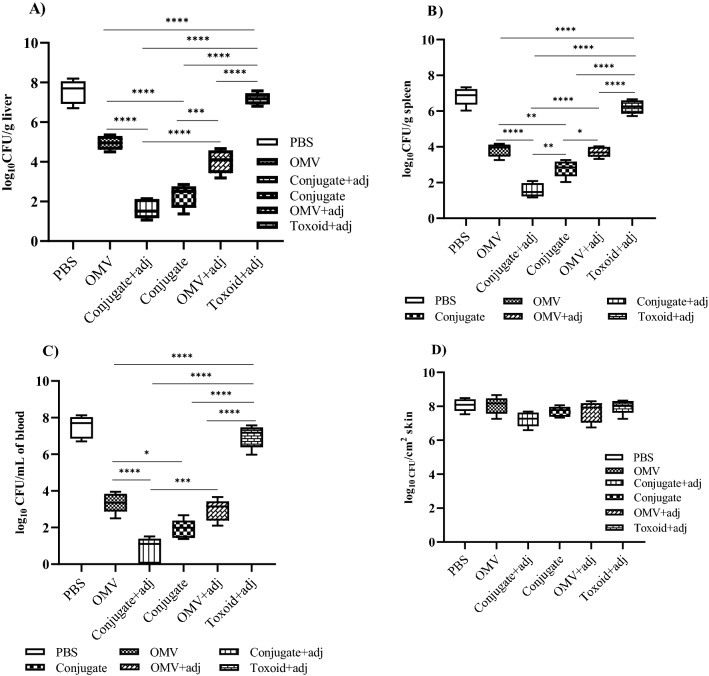
Determination of bacterial load in mice tissues. PA-OMVs-DT + adj reduced the bacterial loads against local and systemic dissemination of PAO1 in mice (n = 5) burn wound infection. Bacterial loads were determined by culturing the liver (**A**) and spleen (**B**), blood (**C**), and skin (**D**) homogenized samples at 24 h, and concentrations were presented in CFU per biopsy sample or gram tissue (CFU/g). Data were presented as box plots, with the median and interquartile ranges indicated. One-way ANOVA was used to compare values. The difference between immunized and control mice groups was indicated as a *p* value. *P < 0.05, **P < 0.01, ***P < 0.001, and ****P < 0.0001 were considered statistically significant.

The bacterial loads in the liver, spleen, and blood of the mice groups vaccinated with conjugated and non-conjugated PA-OMVs, were lower than those in the PBS-administered group (Fig. 4A, B, and C, respectively). The PA-OMVs-DT + adj group had a considerably lower bacterial burden in the liver compared to the other groups, (Fig. 4A) (*P* < 0.0001). Similar findings were seen in spleen samples (Fig. 4B) (*P* < 0.0001). Furthermore, animals immunized with PA-OMVs-DT + adj had a lower bacterial burden in the spleen than mice immunized with PA-OMVs-DT without adjuvant (*P* < 0.05). This was not seen in the liver. The bacterial load in the skin was also reduced in the PA-OMVs-DT + adj vaccinated mice, although the differences were not significant (Fig. 4D).

### Histopathology of PA-OMVs-DT conjugate vaccinated group

**Figure 5:**
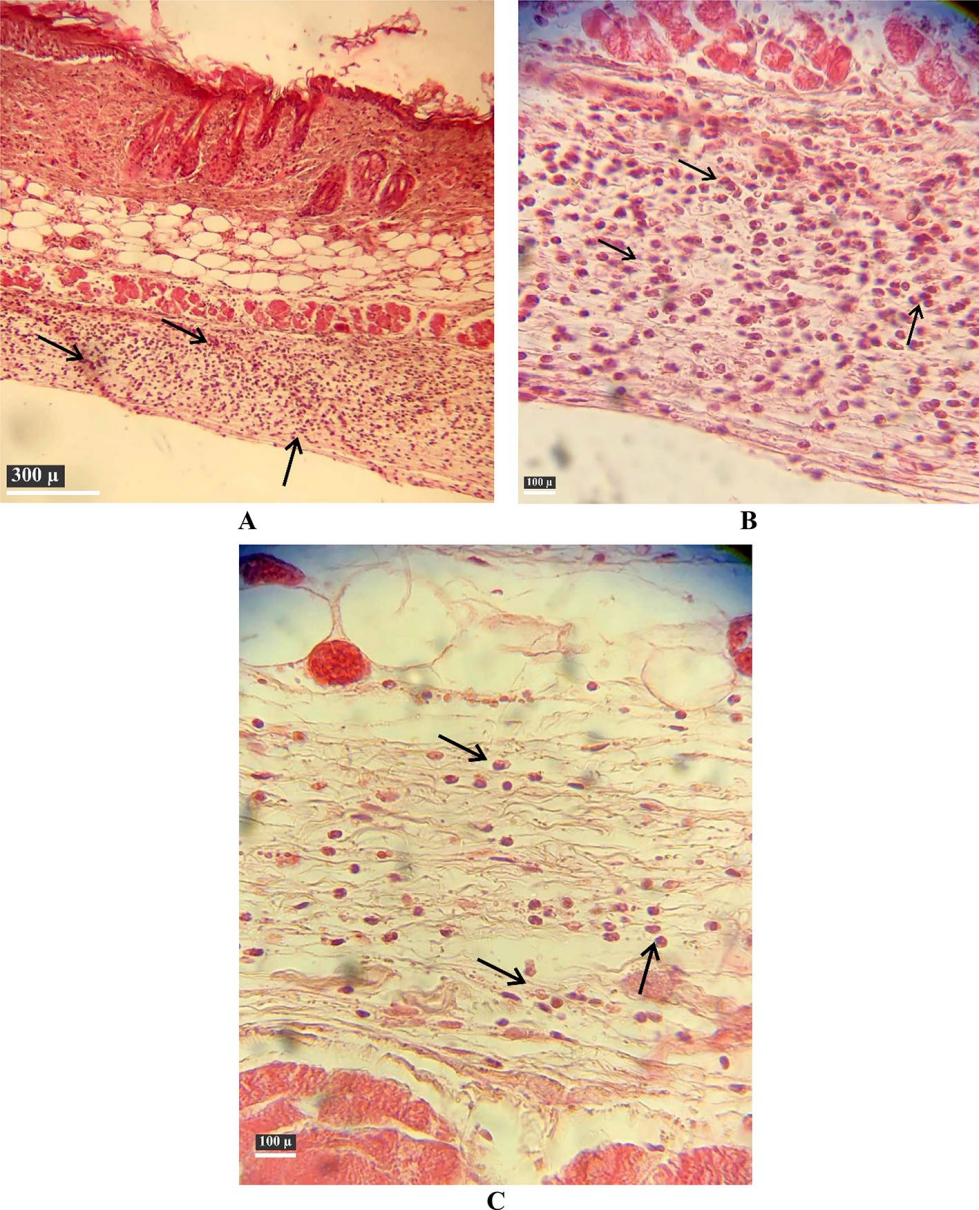
Histopathology of hypodermis. Histopathology analysis showed mild inflammation in the hypodermis of (PA-OMVs-DT + adj) immunized burned mice group. The difference in the histomorphology of burned skin is evident in the H&E-stained sections. In the control group, severe inflammatory cell infiltration is evident in the hypodermis (**A**) (100X), (**B**) (400X). (**C**) The skin of the (PA-OMVs-DT + adj) vaccinated mice revealed eightfold less inflammatory cell infiltration and tissue damage following infection as compared to the control group (400X).

Histological evaluation indicated that the skin structure was disrupted, and the lack of nuclei in the injured tissues confirm a third-degree burn in mice with an average of 94.6 inflammatory cells/mm tissue in quantitative analysis by ImageJ software (Fig. 5A and B). The skin of the (PA-OMVs-DT + adj) vaccinated group exhibited eightfold less inflammatory cell infiltration of 12.6 cells/mm and less tissue damage following infection compared to the PBS group (Fig. 5C).

### Immunization with PA-OMVs-DT + adj conjugate

The ELISA results revealed that there was no change in IgG levels before the first vaccination.

**Figure 6:**
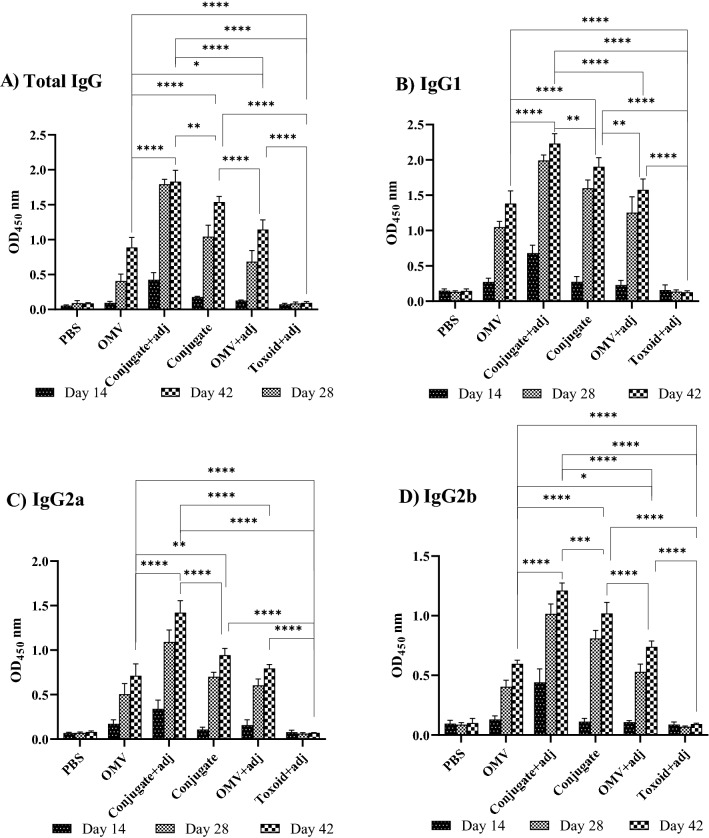
Enzyme-linked immunosorbent assay of IgGs levels raised to antigens. The immunization of mice (n = 5) enhanced the production of specific IgG and subtypes IgG1, IgG2a, and IgG2b responses. (**A**) Specific IgGs levels in immunized groups on days 14, 28, and 42 determined by ELISA. The level of the different IgG subtypes, IgG1 (**B**), IgG2a (**C**), and IgG2b (**D**), were measured from serum samples on days 14, 28, and 42. Data were presented as mean ± S.D. The difference among groups was compared using the two-way ANOVA. *P < 0.05, **P < 0.01, ***P < 0.001, and ****P < 0.0001 were considered statistically significant, respectively.

Two weeks after the last vaccination (day 42), high IgG production was detected in all the immunized mice compared with control mice receiving PBS (Fig. 6A). The PA-OMVs-DT + adj conjugate group produced an IgG antibody level higher than the others (Fig. 6A). The addition of alum to the treatment increased IgG antibody production (*P* < 0.0001) in mice injected with PA-OMVs-DT + adj vs PA-OMVs-DT conjugate without adjuvant. Moreover, in the mice immunized with PBS as a control group, no antigen-specific IgG antibody was detected throughout the immunization process.

The concentrations of specific IgG1, IgG2a, and IgG2b antibodies as markers for Th2 and Th1 responses, were measured. Following the immunization of mice, the concentrations of specific IgG1, IgG2a, and IgG2b antibodies increased in all immunized mice (Fig. 6B, C, and D, respectively). Meanwhile, mice injected with PA-OMVs-DT + adj and PA-OMVs-DT conjugate showed significantly higher IgG1, IgG2a, and IgG2b production compared to other groups (*P* < 0.0001). Mice injected with PA-OMVs-DT + adj conjugate had significantly higher IgG1/IgG2a ratios than non-conjugated groups, with an IgG1/IgG2a ratio of about 1.5.

### Opsonophagocytic killing of the PAO1

**Figure 7:**
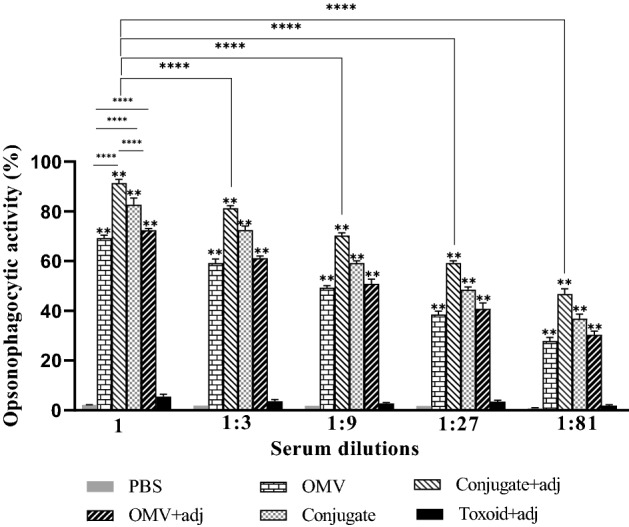
Opsonophagocytic activity against PAO1 in mice immunized with PA-OMVs-DT + adjuvant. Mouse sera from the vaccinated and control groups (n = 5) were pooled to determine the opsonophagocytosis. Multiple comparisons among different groups were analyzed using one-way ANOVA. Data represent the means ± SD of the percentage of killing bacteria phagocytized compared to PBS-immunized samples. **P < 0.01 and ***P < 0.001were considered statistically significant, respectively.

The findings indicate that all the four vaccinated mouse groups had dose-dependent opsonophagocytic activity (Fig. 7). The percentage of killed PAO1 in the PA-OMVs-DT + adj conjugate at dilution 1: 9 (71.3%) (*P* < 0.0001) was significantly higher than that in the PA-OMVs-DT conjugate group (59.23%) (*P* < 0.0001), PA-OMVs + adj group (50.83%) (*P* < 0.0001), and PA-OMVs group (49.2%) (*P* < 0.0001). No apparent opsonophagocytic activity was observed in the PBS group.

## Discussion

OMVs are a potent vaccine candidates for inducing antibacterial protection^32^.

DT was shown to increase the efficacy and protective effect of the vaccine in conjugation with alginate and lipopolysaccharide antigens of *P. aeruginosa*^33^. These findings are consistent with our findings that conjugating a carrier protein (DT) to PA-OMVs can improve the efficacy of OMV as a vaccine candidate.

However, previous studies indicated that OMVs can be used as a carrier protein and in human vaccines in terms of their license^34^. The present study showed better protectivity by PA-OMVs-DT conjugate than the other groups.

Significant components of OMVs from *P. aeruginosa* include OMPs, such as OprF, OprH, and OprG^35^. The protectivity of OMP as a vaccine candidate is well documented^21,36^. Meanwhile, flagellar proteins, composed of type b and c flagellins, have long been considered an ideal vaccine against *P. aeruginosa* infections^37^. Our data indicate that subcutaneouse vaccination of mice with both conjugated and non-conjugated PA-OMVs stimulated the humoral immune system compared to the PBS-administered group. Furthermore, the highest antibody level was observed following third dose. This is in concurrence with a previous study^37^. It has been shown that intramuscular administration of OMVs led to protection against models of *A. baumannii* sepsis and pneumonia^38^. It has been proposed that protection is via bacterial opsonisation^39^, and opsonizing antibodies were induced by intramuscular OMV delivery^38^. This is consistent with the findings of the present study. The evaluation of antibody isotype in mice groups showed that IgG1 was produced more than IgG2a and IgG2b in all immunized mice groups. Since the IgG1 subtype is predominant in the sera of immunized mice, as well as the pattern of IgG1/IgG2a responses, it is possible to explain that humoral immunity is the dominant immune response against *P. aeruginosa* in the burn infection model. Zhang et al. showed that mixed humoral and cellular immune responses were induced, followed by PAO1-derived OMVs immunization as a candidate vaccine^40^. They revealed that humoral immunity might be pivotal for PA-OMVs mediated protection.

Lower bacterial loads in the blood, liver, and spleen of infected immunized mice suggest crucial role of immunization in reduction of the local and systemic dissemination of PAO1 from the infection site. This is further confirmed by histopathology results. Histopathology of skin tissue from the PBS-administered group revealed severe inflammation due to PAO1 subcutaneous injection, whereas immunization with conjugated-PA-OMV-DT + adj reduced inflammatory cell infiltration by eightfold, bleeding, and tissue damage. This may be in terms of the function and presence of the opsonic killing activity of antibodies against PAO1 in the burned wound site, which caused local immunity by decreasing the dissemination of PAO1. Enhancement of humoral immunity by local disruption or elimination of *P. aeruginosa* burden appears to be a priority in overcoming burn infection.

Although induction of IgG antibody titer is a good index of enhanced immunity in defense against bacterial infection, the efficiency and quality of these antibodies are more crucial in the opsonization and phagocytic death of this bacterium. Therefore, to determine the function of an opsonophagocytic of antibodies opsonophagocytosis assay was designed. The findings revealed that specific antibodies promoted opsonophagocytosis and killed PAO1 strain. High titer of opsonic antibodies was induced in mice immunized with non-conjugated and conjugated immunogens. This study showed that the immunization could produce opsonic antibodies that clear PAO1 in a dose-dependent manner, while by increasing the dose, the phagocytosis improves.

One of the most crucial factors to evaluate the efficacy of vaccines is protection against infection challenges. In the mice burn model, enhancement in opsonophagocytosis of the opsonic antibody finally elevates the survival rate of PAO1-infected mice. Our data revealed that vaccination with conjugated and non-conjugated PA-OMVs can protect burned mice against PAO1 infection. Immunization with PA-OMV-DT + adj brought about 100% protection in mice challenged with PAO1. Immunizations with non-conjugated PA-OMVs + adj caused partial protection against infections with PAO1 strain, and the survival rate of mice increased by about 75% compared to the control group. Overall, it seems that OMV-based vaccines could protect burned mice against PAO1 infection.

While the development of OMV-based vaccines looks promising and is currently in its infancy, several challenges remain and need to be considered in further studies, such as the proteomics-based characterization of OMVs, increased yields of OMVs, and decreased LPS toxicity. Our findings indicated the use of conjugated OMVs-DT as a vaccine candidate increased the immunogenicity and protective efficacy against PAO1 in burn wound infection mice. In conclusion, our study provided the basis for subsequent studies into preventing *P. aeruginosa* infection in burn wounds by immunization and paved the way for further investigation. Mass production of OMVs, and concerns associated with the LPS toxicity are the potential hurdles or limitations of this study to a viable clinical product, that need to be resolved in further studies. These findings support further development of OMVs as a vaccine platform against *P. aeruginosa* and warrant further exploration of intranasal delivery as a route of immunization. Vaccine delivery route is an important consideration for vaccine development^41^.Mucosal vaccinations elicit good local mucosal and systemic immune responses and would be easier to administer than injectable vaccines, resulting in a decreased risk of infection^42,43^. Mucosal immunization may also lead to qualitatively better immune responses, for example in humans intranasal immunization induced upper airway IgA responses^42^.

## Materials and methods

### Approval for animal experiments

All experimental protocols were approved by the Ethics Committee of Shahed University (Tehran, Iran) vide approval certificate reference IR.SHAHED.REC.1398.057. All methods were carried out following relevant guidelines and regulations of the National Institute of Health guide for the care and use of laboratory animals (NIH Publication No_ 8023, revised 1978). We confirm that this study is reported in accordance with ARRIVE guidelines. Female BALB/c mice aged six to eight weeks were purchased from Razi Vaccine and Serum Research Institute. Mice were matched for age and sex, maintained under the same settings (12:12 h light/dark cycle, 22–23 °C, and 40% humidity) throughout the trial, and kept in specific pathogen-free (SPF) environments. All mice were fed with a standard antibiotic-free diet and water ad libitum.

### Purification and characterization of PA-OMVs

OMVs isolation and purification from *P. aeruginosa* were, according to Siadat et al.^28^. Briefly, *P. aeruginosa* strain PAO1 was obtained from Molecular Microbiology Research Center of Shahed University, Tehran-Iran. The bacterial culture in 1 L of LB broth was maintained at 37 °C, shaking at 200 rpm to an optical density of 1.2. PAO1 cells were centrifuged at 6000 rpm for 30 min at 4 °C. The pellet was stabilized in a volume 7.5 times its wet weight with a 1.0 M Tris buffer containing 10 mM EDTA (w/v). The suspension was again centrifuged at 20,000 g for 1 h at 4 °C. The suspension was supplemented with a volume of 1:20 of 0.1 M Tris buffer solution containing EDTA and 100 g/L sodium deoxycholate. After 10 min, the pellet was suspended in deoxycholate; then, it was separated by ultracentrifuge at 125,000 × g for 2 h at 4 °C. The PA-OMVs were filtered through 0.22-mm filter (Millipore, USA) and stored at − 70 °C for further use. The total protein concentration of PA-OMVs was measured using the Nanodrop and Bradford assay. The protein sample pattern was evaluated on SDS-PAGE. Ultimately, the LAL assay (Lonza, Walkersville, MD, USA) was used to determine the amount of endotoxin.

### Field emission scanning electron microscopes (FE-SEM)

The size and shape of extracted PA-OMVs were evaluated by FE-SEM (MIRA3 TESCAN FESEM, Czech Republic).

### Conjugation of OMVs-DT

PA-OMVs were covalently conjugated to DT as a carrier protein using the adipic acid dihydrazide (ADH) (Sigma, USA) as a spacer molecule and 1-ethyl-3- (3- dimethyl aminopropyl) carbodiimide (EDAC) (Sigma, USA) as a linker. At first, 2 mg/ml of PA-OMVs was exposed to EDAC (final concentration of 0.1 M), and then reacted with ADH for 5 h at pH: 8. Thin-layer chromatography (TLC) was used to monitor the reaction's solution for 6 h. The conjugation of PA-OMVs and ADH was then purified for 24 h using three water changes and a 2 kDa cut-off dialysis bag against water at 4 °C. Then, 2 mg/ml of this solution reacted with DT containing 1 mg/ml protein in the presence of EDAC for 24 h. Then, PA-OMVs-DT conjugate was purified via Sephadex G-750 gel filtration chromatography (Sigma, USA).

The fractions were collected and centrifuged as a PA-OMVs-DT conjugate after being measured at 280 nm (protein) in the obtained fractions. Finally, the conjugated molecules were lyophilized in glass vials with a volume of 1 ml, passed through a 0.45 mm Millipore filter (USA), and kept at − 20 °C until injection.

### Characterization of PA-OMVs-DT conjugate

The conjugated PA-OMVs-DT samples were analyzed using a Fourier transform infrared (FTIR) spectroscopy (Bruker, Germany), Mapping، FE-SEM (Zeiss, Oberkochen, Germany), and Energy dispersive X-ray spectroscopy (EDAX) (Oxford Instruments).

### Immunization of mice groups

The mice were divided into six groups; group I was injected with sterile PBS. Mice within group II received PA-OMVs (50 µg/mL), group III received conjugation of OMVs-DT formulated with alum adjuvant (Sigma, USA) (PA-OMVs-DT + adj), group IV received the conjugation of PA-OMVs-DT, group V received PA-OMVs formulated with alum adjuvant (PA-OMVs + adj), group VI received DT formulated with alum adjuvant (DT + adj), on days 0, 14 and 28. All the mice groups were immunized subcutaneously (SC), injecting 100 µl in groups without alum adjuvant and 200 µl in groups with alum adjuvant. Blood was obtained from the orbital sinus on days 0, 14, 28, and 42, and serum was collected for further analysis. The Control group consisted of non-immunized, non-infected burn mice.

### Development of burn infection

Two weeks after the last immunization, on day 42, the immunized mice were burned and challenged^36^. In brief, to create a burn, the back portions of the mice in each group were shaved at least 24 h before burn wound induction. Then, mice were anesthetized with the anesthetic drug Ketamine (100 mg/ml) and Xylazine (20 mg/ml) mixture. A third-degree burn wound was created by a custom-made cylindrical probe (20 × 20 × 100 mm, 150 g) heated by a gas flame to the temperature of 104 °C and put on the shaved part of the animal for 8 s. Immediately after burning, 500 μl of 0.9% saline was injected intraperitoneally (i.p.) into the burned mice for fluid replacement. Acetaminophen (0.25 mg/ml) was used as post-burn analgesic. Subsequently, the mice were infected by subcutaneous injection of a lethal dose (3 × 10^2^ CFU) PAO1 at the burn center.

### Enzyme-linked immunosorbent assay (ELISA)

The specific antibodies in immunized mice were analyzed using an indirect ELISA assay^44^. Briefly, plates were coated with 100 µl of purified PA-OMVs (50 µg/mL) in 50 mM carbonate buffer (pH: 9.7) (5 µg/well) overnight at 4 °C. Then, the mice sera were diluted at 1:100, 1:600, 1:1200, and 1:36,000 in PBS. The secondary antibodies were goat anti-mice conjugated to horseradish peroxidase (Sigma, St. Louis, USA), anti-IgG, IgG1, IgG2a, and IgG2b (Sigma). The absorbance was read at 450 nm. The negative control was serum from mice that had been vaccinated with PBS.

### Determination of bacterial burden and survival rate

Twenty-four hours after infecting the burnt wounds with 3 × 10^2^ CFU of PAO1, the immunized and control mice groups were sacrificed. To determine the local dissemination of PAO1 strain in burn wound infections, burned skin at the injection site (15 × 15 mm) was collected. Moreover, to detect the systemic dissemination of PAO1, the infected mice's liver, spleen, and blood were aseptically collected. After that, the tissues were weighed, and sterile PBS was used to homogenize them. Then, homogenous samples were serially diluted in sterile PBS before being plated on Nutrient Agar (NA) and incubated for 24 h at 37 °C. The number of colony-forming units (CFUs) from each plate was then measured as CFUs per gram of tissue (CFU/g). The survival rate of experimental mice in each group was monitored for a 10-day.

### Histological analysis

The skin samples were collected and fixed in 10% formalin, then embedded in paraffin, stained with Hematoxylin and Eosin (H&E), and observed under a light microscope. ImageJ is a well-known and publicly available image processing tool (http://rsbweb.nih.gov/ij/) released with many plugins and macros useful to biomedical image processing^45^. Automatic mammalian cell counting with ImageJ was previously reported^46^. In this study, an automated counting method utilizing ImageJ 1.53t (https://imagej.nih.gov/ij/download.html) was used for quantitative analysis of inflammatory cell infiltration. Five field images of each histogram showing various concentrations of inflammatory cell infiltration were studied.

### Opsonophagocytic assay

An opsonophagocytic assay was performed as previously described^47^ with slight modification. In brief, PAO1 strain was grown in TSB and incubated at 37 °C until the OD650 reached 0.20. Mouse macrophages were adjusted to 2 × 10^7^/ml in RPMI-1640 (Gibco, Germany) supplemented with 10% heat-inactivated fetal bovine serum (FBS). Fresh infant rabbit serum (Institute Pasture, Karaj, Iran) served as a complement source. Sera samples from immunized mice containing opsonic antibodies were heat inactivated (56 °C, 30 min) and serially diluted. These components were mixed for the opsonophagocytic test in a sterile microcentrifuge tube containing 100 µl of each component, and then incubation was carried out at 37 °C for 90 min in a shaker. Negative controls included 100 µl of RPMI-1640 medium/ FBS instead of antibodies, complement, and macrophages in each assay. After 90 min incubation, 50 µl aliquot was removed, diluted in PBS containing 0.05% Tween 20, and grown on TSA. The percent opsonic activity of immune serum was calculated as follows:

Opsonophagocytic activity (%) = [1 − (CFUs for immune serum at 90 min/CFUs for preimmunize serum at 90)] × 100.

### Statistical analysis

All experiments were performed in triplicate for each sample, and the results are expressed as mean ± standard deviation (SD). SPSS 24.0 (SPSS, Inc., USA) and GraphPad Prism version 9.4.1.681 (GraphPad Software, Inc., USA) were used to conduct statistical analyses and comparisons. A two-way analysis of variance (ANOVA) was used to assess the ELISA assays. Tukey’s multiple comparison test was used. The one-way analysis of variance (ANOVA) used to analyze bacterial loads and opsonic killing action. The survival data were analyzed by Kaplan–Meier survival curves and log-rank test. *P* values < 0.05 were considered statistically significant (“Supplemnatary information”).

## Supplementary Information

Supplementary Information.

## Data Availability

The datasets generated and/or analyzed during the current study are available from the corresponding author upon reasonable request.
