# Supplementary figures and images for: Pseudomonas aeruginosa PAO1 outer membrane vesicles-diphtheria toxoid conjugate as a vaccine candidate in a murine burn model
Source: Sci Rep. 2022 Dec 24;12:22324. doi: 10.1038/s41598-022-26846-z (PMC9789887; doi:10.1038/s41598-022-26846-z)

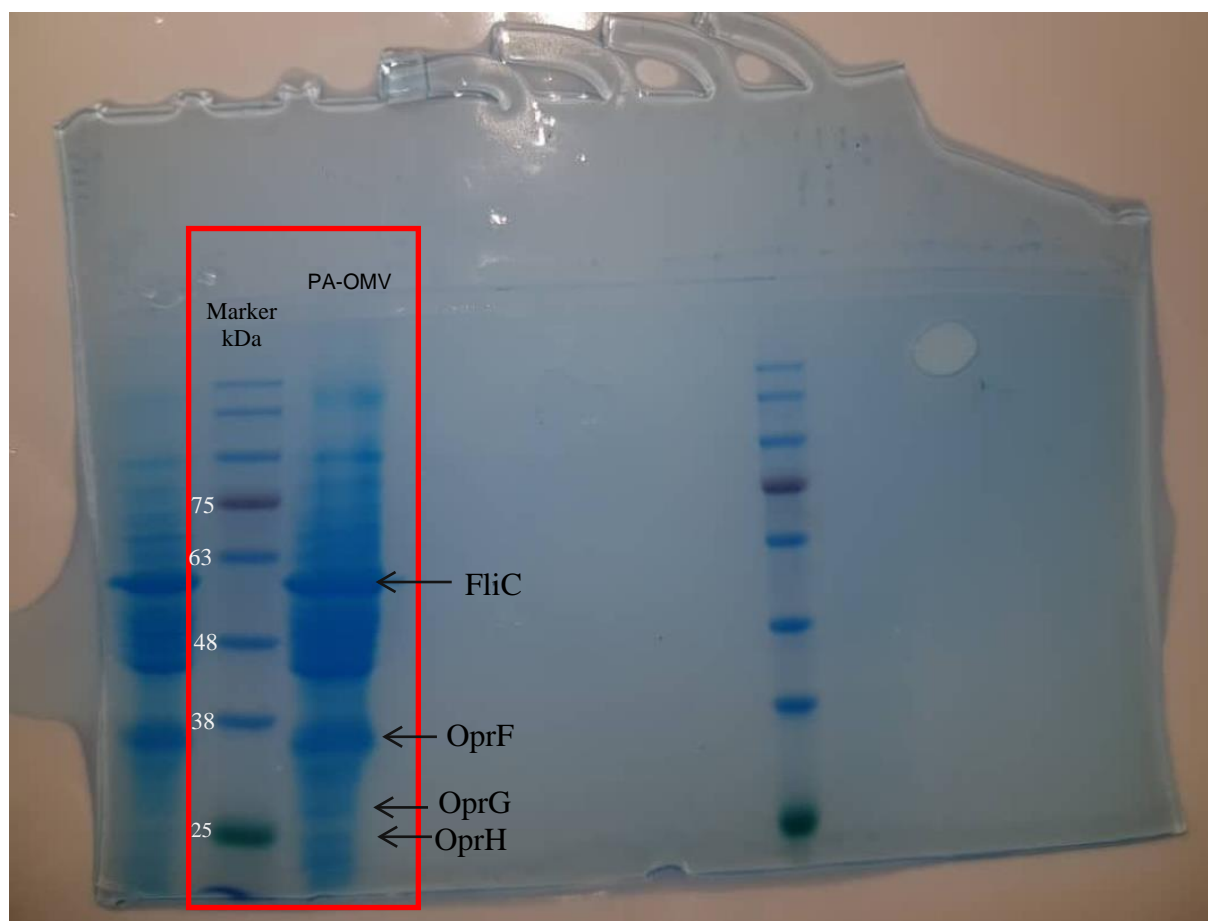

**Supplementary Figure 1**

Coomassie brilliant blue-stained SDS-PAGE of PA-OMVs

Supplement: Supplementary file 1 — Supplementary Information. [file 41598_2022_26846_MOESM1_ESM.pdf]
